# Children’s cortical speech tracking in face-to-face and online video communication

**DOI:** 10.1038/s41598-025-04778-8

**Published:** 2025-06-20

**Authors:** Fatih Sivridag, Josefine Schürholz, Stefanie Hoehl, Nivedita Mani

**Affiliations:** 1 University of Göttingen, Psychology of Language, 37073 Göttingen, Germany; 2 Leibniz Science Campus Primate Cognition, 37077 Göttingen, Germany; 3 University of Vienna, Faculty of Psychology, 1010 Vienna, Austria

**Keywords:** Human behaviour, Language

## Abstract

In today’s digital age, online video communication has become an important way for children to interact with their social partners, especially given the increased use of such tools during the pandemic. While previous studies suggest that children can learn and engage well in virtual settings, there is limited evidence examining the neural mechanisms supporting speech processing in face-to-face and video interactions. This study examines 5-year-old German speaking children’s cortical speech tracking (n = 29), a measure of how their brains process speech, in both scenarios. Our findings indicate comparable levels of cortical speech tracking in both conditions, albeit with subtle differences. This implies that children exhibit similar neural responses to speech in both situations and may adopt different strategies to overcome potential challenges in video communication. These neural results align with previous behavioural findings, supporting the notion that live online video interactions can serve as an effective communication medium for children.

## Introduction

Virtual interactions that are established via a screen and headphones are replacing many types of face-to-face (f2f) interactions and are gradually becoming the norm. Children’s interactions with their peers and adults are increasingly shifting from being in-person to relying on digital media, both in their leisure activities and school experiences. These technologies have become widely available to the general public only relatively recently and the momentum of their adoption has significantly escalated due to the global pandemic^1^. Thus, recent data suggests a 32% increase in children’s screen time in the last decades^2^, with children of 4–6 years of age having access to between 20 and 240 min of daily screen time across six different European countries^3–5^. Research on their effectiveness has not, however, been able to keep up with the rapidly increasing access to digital media in young children. Although there are behavioural studies suggesting that children can follow and learn from live online interactions to an extent that is comparable to their performance in f2f communication^6–10^, there is not much evidence about the neural mechanisms underlying such observations. Against this background, the current study examines cortical speech tracking in f2f interactions relative to naturalistic live digital interactions—akin to commonly used video conferencing tools—in young children between 5 and 6 years of age.

Online video communication lacks some of the affordances that f2f communication has by default. One important difference is the synchrony between visual and auditory streams of information. While, in f2f communication, the lag between the two streams is constant and usually negligible, in video communication, this lag dynamically changes to the magnitude of seconds depending on several technical factors^11^. Moreover, while locating the source of sound in the environment is an automatic process and guides attention, in video communication, the auditory input always comes from the same fixed source (i.e., speakers or headphones) which strips the auditory signal of highly relevant localization information. Video communication also has limited affordances with regard to eye contact^12^ and joint attention between the speaker and the listener. Signaling mutual gaze and directing the attention of the other to a certain location using gaze are both less precise and more difficult in video communication^13^, while they are automatic processes in f2f conversations due to high salience of gaze^14^. Relatedly, common ground between the speaker and the listener is usually missing in video communication^15^. In particular, while it can be achieved to some extent with screen sharing, it is restricted to two-dimensional space and the objects on the screen cannot be pointed at or referred to using salient ostensive cues like gaze or declarative pointing. The lack of senses such as touch and smell is yet another missing affordance of video communication.

Despite the lack of these affordances and relatively poor quality of audio and visual input compared to the f2f interactions, online video communication still allows bidirectional contingent interactions that can closely resemble f2f interactions. People can efficiently communicate over online videos in a wide variety of contexts. Indeed, several studies have shown that children too can engage socially and learn effectively through online video. For example, Roseberry et al.^8^ found that toddlers learned new verbs equally well in f2f and online video interactions, highlighting the importance of social contingency. Similarly, Myers et al.^16^ showed that online video facilitated both social bonding and word learning in young children. Other studies found that children were more successful in tasks like imitation^7^ and object retrieval^10^ when these were presented via live rather than pre-recorded video. Although some studies report mixed results and emphasize the moderating role of factors such as parental support and task difficulty^17,18^, the overall evidence supports the notion that online video communication, while lacking some sensory and contextual affordances, can successfully support language learning and social interaction in early childhood.

Although behavioural evidence implies that children can interact with others and learn language efficiently in online video communication, there are not many studies examining the neural mechanisms supporting children’s speech processing during f2f and online video communication. One phenomenon, referred to as cortical speech tracking, is suitable for revealing some of the potential differences in the neural response to speech in different media. This technique exploits the correlation between the physical properties of an auditory stimulus, such as the speech envelope, which represents the variation in sound amplitude over time, and the brain’s cortical electrical activity. Cortical speech tracking is fairly robust in probing the mechanisms that enable the brain to selectively attend to relevant sources of information and filter out noise in the stimuli, thus ultimately leading to better understanding of top-down and bottom-up processing pathways^19^, which have been suggested to work in tandem in speech processing^20^.

Cortical speech tracking has also been observed with children in a number of studies (e.g., Jessen et al.^21^). Most of these studies have examined the extent to which infants’ neural activity tracks infant directed speech (IDS) better compared to adult directed speech (ADS)^22–25^. However, there are few studies examining cortical speech tracking in older children. In one cross-sectional study, enhanced cortical tracking of audiovisual speech, which refers to speech accompanied by visual elements such as lip movements, was observed with infants and adults, but not with 4-year olds^25^. The authors noted that the lack of audiovisual enhancement of cortical speech tracking in 4-year-olds was unexpected and attributed this result to the use of IDS as their stimulus. In other words, they suggested that 4-year-olds may have prioritised auditory information to process the simplified speech signal, leading to reduced enhancement of cortical speech tracking from the visual input. Vander Ghinst et al.^26^ compared cortical speech tracking between 6- to 9-year-old children and adults and found that children’s cortical speech tracking decreased more dramatically compared to the adults when noise was added to the speech. However, similar to adults’, children’s cortical activity tracked the attended speech stream in multi-speaker contexts and similar brain areas contributed to cortical speech tracking in both groups. Together, these findings support cortical speech tracking as a robust and informative measure of speech processing in children across different ages.

Although there are no studies using cortical speech tracking to directly compare f2f and video communication to the best of our knowledge, research on cortical speech tracking in sub-optimal situations, such as Cocktail Party situations^27^ or when the speech is less intelligible^28^ can help inferences about the speech processing in online video communication. In Cocktail Party studies, in which participants hear two different speakers at the same time, neural responses track only the envelope of attended speech^29^. This finding suggests that attention has a role in cortical speech tracking, especially in sub-optimal situations^30,31^.

At the same time, somewhat counterintuitively, while the addition of noise decreases speech intelligibility, cortical speech tracking is enhanced in noisy conditions^28,32,33^. For instance, Herrmann^32^ has shown that cortical speech tracking is enhanced when speech is masked by background noise. Herrmann attributes this effect to stochastic resonance, where adding noise to the system improves the response of the system to a particular input source, due to noise enabling near-threshold neurons to fire. Vanthornhout et al.^33^ also found higher cortical speech tracking in low signal-to-noise ratio settings and highlighted the role of attention in cortical speech tracking in noisy conditions. They suggested that in the absence of noise, listeners tend to rely on passive listening, which requires minimal attentional effort. In contrast, noisy situations demand more attention and, consequently, engage increased neural resources. Similarly, Yasmin et al.^28^ observed a U-shape relationship between noise and cortical speech tracking, such that cortical speech tracking was at the highest level when the noise level was moderate and it decreased when the signal-to-noise ratio decreased or increased. The authors also found that the latency of peak cortical speech tracking increased as the level of noise rose. They suggested that when speech is mixed with noise but remains intelligible, comprehension requires more effort, resulting in stronger cortical speech tracking. Additionally, they proposed that noisy environments slow the processing of acoustic features, causing the peak in cortical speech tracking to emerge later as the signal-to-noise ratio decreases.

Most previous studies, which have investigated cortical speech tracking in different settings, presented participants with exactly the same auditory and visual input in highly controlled laboratory settings to eliminate potential confounding effects of stimulus properties on cortical speech tracking^21,34^. Alternatively, previous work has examined processing of pre-recorded, idealized audiovisual stimuli-typically close-up videos of isolated speakers with clear, noise-free audio^25,35,36^ (but see Menn et al.^24^). Additionally, many studies use very short stimuli which might minimize or eliminate semantic content to suppress top-down effects, which may underestimate how meaningful and engaging content influences cortical speech tracking^19,30^. While such setups help control for confounding variables, they fail to capture the variability and imperfection of real-life communication-whether f2f or over video-where audiovisual signals are rarely perfectly synchronised and distortions are common. In online video communication, in particular, temporal mismatches and signal degradation can make visual cues less reliable due to the factors like limited bandwidth, packet loss, and latency.

The assessment of cortical speech tracking in real-time, naturalistic f2f and video communication, in contrast, has the potential to offer new insights into cortical speech tracking and speech processing in ecologically valid contexts^37^. Unlike traditional experimental setups, these naturalistic interactions involve continuous bidirectional verbal and visual exchanges, spontaneous adjustments to the speech based on the non-verbal feedback from the listener, and multimodal cues, such as facial expressions and gestures. These social elements may play a crucial role in shaping cortical speech tracking, potentially facilitating alignment between auditory and visual signals in a way that is not captured in highly controlled experimental conditions. Taken together, it would be valuable to examine how previous laboratory findings on cortical speech tracking translate to more naturalistic and socially meaningful communicative settings—especially in comparison of f2f and video communication.

Against this background, in this study, we examined 5-year-old children’s cortical speech tracking to age-appropriate stories in f2f and live online videos. We recruited 5- to 6-year-old children and recorded brain activity using EEG while they listened to stories told by an adult story-teller. Each child listened to a story in the f2f condition, where the adult sat in front of the child in the same room as the child and told them a story. The same child also listened to a different story in the video condition; where the story-teller was in a different room and told the story to the child via a video conferencing tool running on a tablet device.

Complete methodological details, including details of EEG analyses, are presented in the Methods section. In brief, we used a Temporal Response Function (TRF) to examine the extent to which the envelope of the speech heard by the children was represented in the EEG signal. TRF is a popular operationalization of cortical speech tracking, in which a model is trained to define how certain features in the stimulus are represented in the neural response (encoding or forward models) or vice versa (decoding or backward models)^38^. Here, we modelled cortical speech tracking using a subject-specific forward Temporal Response Function (TRF) approach, where regularized regression was used to estimate the relationship between the speech envelope and EEG signals. The data were segmented into equal-length trials for each participant, and model prediction accuracy﻿—correlation between predicted and actual EEG﻿—served as the outcome measure. The study and hypotheses have been pre-registered on Open Science Framework Platform (https://osf.io/yucd7).

Our research questions (RQ) and hypotheses (H) are as follows:

- **RQ1.** Do children show cortical speech tracking in both the f2f and video conditions?
  - **H.1.** Children will show cortical speech tracking in both f2f and video conditions as indexed by significantly improved neural response prediction compared to a random distribution.
  Evidence from earlier studies shows that cortical speech tracking is quite robust and emerges even when the speech is noisy^38^. Furthermore, naturalistic interactions, regardless of medium, provide key communicative affordances that facilitate cortical speech tracking. Previous studies have demonstrated that verbal and non-verbal social cues can enhance language processing and learning^7,8,10^. In both live f2f and online video settings, these social cues remain present, distinguishing them from prerecorded videos or audio-only conditions. We anticipate, therefore, that the continuous exchange of communicative signals will support robust cortical speech tracking in both conditions, despite potential differences in sensory affordances (e.g., depth perception in f2f interaction vs. flat screen presentation in online interaction).
- **RQ2.** Are there quantitative and qualitative differences in cortical speech tracking between f2f and video communication?
  - **H.2.1.** Cortical speech tracking will be enhanced in the video condition compared to f2f condition.
  We expect cortical speech tracking to be enhanced in the video condition relative to the f2f condition. On the one hand, this may seem counterintuitive due to the lack of affordances in video communication relative to f2f communication discussed above. On the other hand, as highlighted above, a number of studies report finding increased cortical speech tracking in noisy settings^28,32,33^. Such findings are typically explained either by the increased attentional demands introduced by noise^33^ or due to stochastic resonance^32^. In our study, the video condition introduces more environmental noise and potential misalignments between audio and visual streams compared to the f2f condition due to limited bandwidth, inherent variability in synchrony between audio and video, and lack of affordances. These features may prompt listeners to engage more effortfully with the speech and attend to the speech more actively in online video condition compared to f2f condition, where more passive listening might be sufficient for comprehension. As attention plays a key role in modulating cortical speech tracking, especially in challenging listening environments^28,33^, we anticipate higher levels of cortical speech tracking in the video condition.
  - **H.2.2.** Cortical speech tracking will emerge with greater time delays, i.e., with longer time shifts between EEG and audio, in online video condition compared to f2f condition.
  Previous research suggests that when the speech is noisy, peak cortical speech tracking emerges with greater time delays due to increased processing demands^28^. In particular this work suggests that, while noisy conditions may engage more neural resources, thereby resulting in stronger cortical speech tracking (H.2.1.), such conditions may nevertheless slow processing of input, leading the peak in cortical speech tracking to emerge later. In keeping with this work, we predict that peak cortical speech tracking will emerge with greater time delays between speech and EEG signal in the video condition relative to the f2f condition. Furthermore, online video communication does not provide reliable visual cues such as lip movements due to the low image resolution and possible lags between audio and video. Lip movements normally precede the sound by 100–300 ms^39^, and they are usually utilized to predict upcoming words. Indeed, being able to see the face of the speaker has been shown to speed up speech processing^34^. Therefore, as need for processing resources increases due to noise and lip movements becoming unreliable, we anticipate cortical speech tracking to emerge with greater delays in online video condition compared to f2f condition.

## Results

We analysed children’s responses to the questions, which were asked while they were listening to the stories, to see if they were attending to the stories throughout the experiment. Overall, children responded to 83% of the questions correctly. A two-tailed t-test did not show any significant difference in the correct answers between the f2f and online video conditions (*t*(142) = 0.91, *p* = 0.36). In other words, we found no evidence for a difference in children’s attention and responses to the stories across the two conditions.

The mean and the standard deviations of prediction accuracies with different time delays for both conditions are presented in Table 1. Prediction accuracies were binned and averaged for each 50 ms delay for display purposes, although time delays increased in steps of 7.8 ms in the analysis.

**Table 1:** Mean and standard deviations of prediction accuracies across conditions and time delays

Time delay ms	f2f		Video	
	Mean	sd	Mean	sd
0–50	0.0074	0.027	0.0123	0.027
50–100	0.0084	0.024	0.0108	0.028
100–150	0.0093	0.026	0.0109	0.027
150–200	0.01	0.027	0.0106	0.028
200–250	0.0101	0.027	0.013	0.028
250–300	0.0096	0.027	0.009	0.027
300–350	0.0090	0.026	0.0069	0.027
350–400	0.0082	0.026	0.0050	0.027
400–450	0.0076	0.025	0.0041	0.027
450–500	0.0066	0.024	0.0029	0.026
500–550	0.0061	0.023	0.0016	0.025
550–600	0.0047	0.025	0.0002	0.024

**Fig. 1:**
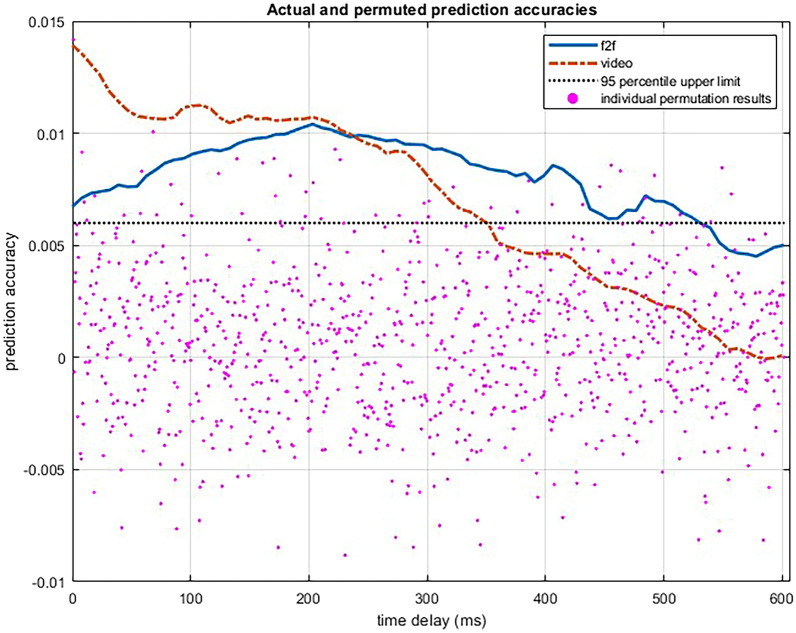
Actual and permuted prediction accuracies. Dotted line shows the upper limit of 95% percentile of permuted prediction accuracies. (f2f: face-to-face).

Permutation on the randomly paired EEG and speech envelope data returned a random distribution of prediction scores ranging between $-0.009$ and 0.014, with 95% of the prediction scores being lower than 0.006. Comparison of actual prediction accuracies with the random distribution revealed that, in the f2f condition, prediction accuracies were greater than the 95% of the random distribution between 0 and 523 ms time delay (Fig. 1). In the video condition, prediction accuracies were higher than 95% of the random distribution between 0 and 343 ms time delay. These results confirm our first hypothesis (H.1.) that children show cortical speech tracking while listening to stories in both f2f and online video conditions.

**Fig. 2:**
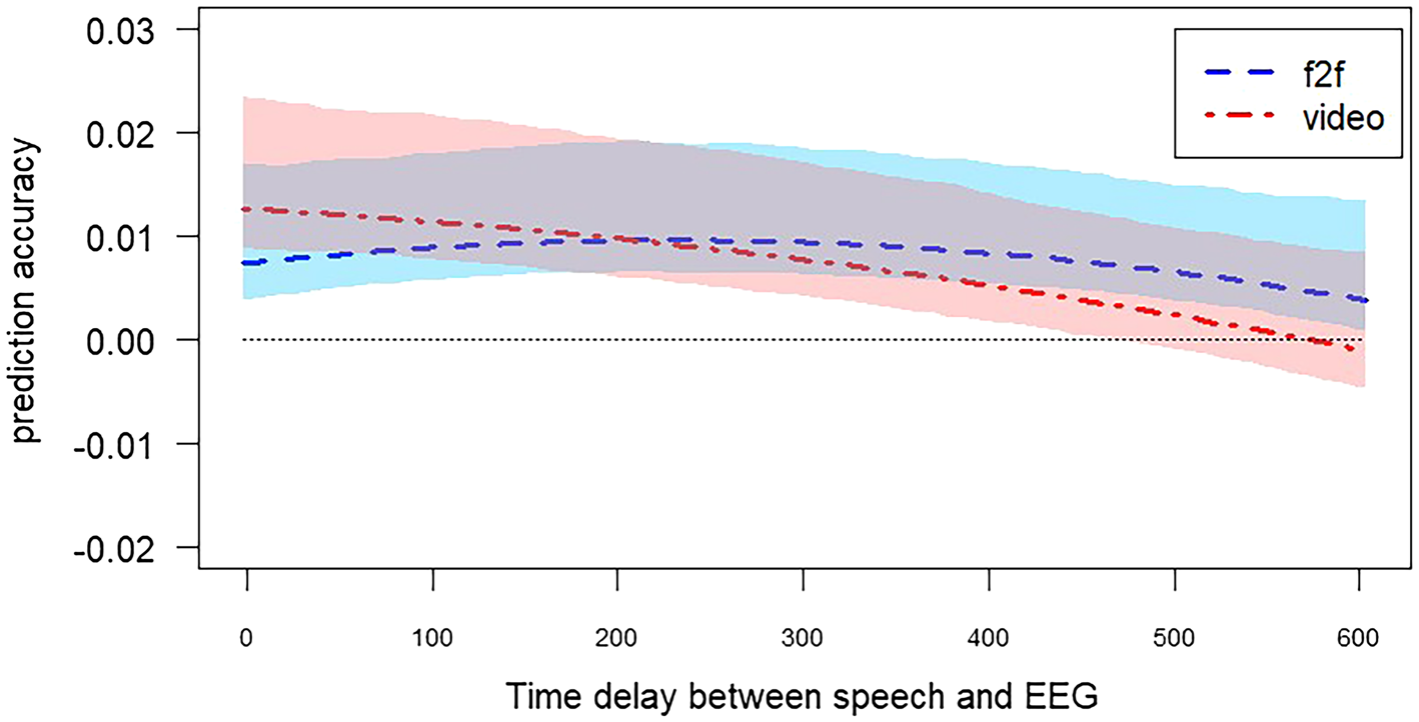
Fitted values obtained from generalized linear model. Shaded areas show 95% confidence intervals for fitted values based on bootstrapping results. The dotted line corresponds to 0. (f2f: face-to-face).

Our generalized linear mixed-effects model to test whether there were any significant differences in the magnitude and time course of cortical speech tracking between the f2f and video condition revealed only a significant effect of time ($\beta _{time} = -0.008$, *p* = 0.047), such that the amplitude of cortical speech tracking decreased with increasing latency between speech and neural signal. Condition ($\beta _{condition=video}$ = 0.003, *p* = 0.724), quadratic time term ($\beta _{time^2} = -0.001$, *p* = 0.568), and two-way interactions between condition and time ($\beta _{condition:time}$ = 0.006, *p* = 0.205) and condition and quadratic time ($\beta _{condition:time^2} = -0.001$, *p* = 0.597) did not have any significant effect on prediction accuracies. Fitted values and 95% confidence intervals obtained from the model are shown in Fig. 2. Comparison of the full model with a reduced model, which did not include the predictors of interest (condition, time, and quadratic time) and their interactions, also yielded a non-significant result ($\chi ^2$(5) = 4.29, *p* = 0.5) showing that our full model does not explain more variance in the data compared to a model without the predictors of interest.

A significant negative estimate for time was expected given that we used relatively large time delay (0–600 ms) in our analysis and cortical speech tracking is expected to diminish after a certain time delay as brain continues with processing new stimuli^40^. Non-significant effects of condition, quadratic time, and two-way interactions suggest that there was no evidence for differences in prediction accuracies across the two conditions, i.e., that there was no difference in cortical speech tracking in the f2f and the video condition (H.2.1.). Although Fig. 1 implies that with shorter time delays, prediction accuracy is higher for the video condition, this effect does not reach significance. As stated in H.2.2, we also predicted a significant interaction between time delay and condition, such that we predicted that prediction accuracy would reach its peak later in the video condition compared to the f2f condition. This two-way interaction was not significant. Moreover, Fig. 1 implies an opposite trend of what we were expecting. Although statistically not significant, it appears that, in the video condition, peak cortical speech tracking emerges with almost no time delay while it takes around 200 ms in the f2f condition for cortical speech tracking to reach its peak.

## Discussion

In this study, we examined children’s cortical speech tracking while they were listening to stories told either face-to-face (f2f) or through live online video communication. Our results show that children, indeed, show significant cortical speech tracking in both conditions, a finding we expected to observe. However, contrary to our expectations, there were no significant differences in the magnitude and time course of cortical speech tracking across the two conditions. Moreover, the results suggested that, in the video condition, neural response prediction accuracy, which is a measure of cortical speech tracking, seemed to be more successful with shorter time delays, albeit not significant. These results have several implications in terms of the use of online video as a communication tool especially in educational domains.

Although cortical speech tracking was expected in both conditions (H.1.), we had anticipated that it would be higher (H.2.1.) but emerge with increased time delays between speech and EEG signal in video communication compared to f2f condition (H.2.2.). However, we did not find any significant differences in the magnitude or time course of cortical speech tracking across conditions. This indicates that despite the dynamic temporal, spatial, and spectral noise in video, children’s neural activity, as measured here, can track the speech similarly in both media of communication—bearing in mind that f2f settings may also involve other types of distraction (e.g., environmental or social cues). Taken together with previous findings that cortical speech tracking emerges even when the speech is convoluted with different types of noise^26,28,31^, this finding shows that cortical speech tracking is also robust to the type of noise which is inherently present in online video communication. This result is also in line with the findings of previous behavioural studies, which have shown that children can understand and follow instructions^10^, imitate others^7^, and learn words^8,9^ from video communication.

We had reasoned that the online video condition would require higher attention and listeners would engage with the speech processing more actively and effortfully due to the noise. If this were to be the case, we might expect the children to try to get as much information from the available cues as possible to compensate for the loss due to the noise, i.e., low quality of audio and video. Indeed, a behavioural analysis of gaze data of a subset of our sample (n = 8) for another project revealed that children spent a greater proportion of time looking at the face of the speaker in the video condition compared to the f2f condition ($\hbox {Median}_{f2f}$ = 0.33, $\hbox {Median}_{video}$ = 0.55, *z* = 5, *p* = 0.023; Wilcoxon signed-rank test; see supplementary material A for details). Although speculative, this increased attention to the speaker’s face in the video condition may be taken as evidence that children were trying to get as much information from the speaker’s face as possible in the video communication, showing that video communication requires more active and effortful listening.

Although we did not find support for our hypotheses about the differences in the cortical speech tracking between the two conditions, our results are still interesting as they suggest that children process speech similarly in both media of communication. A potential explanation for the lack of difference in cortical speech tracking between the two conditions comes from the contextual cues included in the stories presented to children. In particular, the stories we used were age-appropriate and child-friendly. Although our participants had not heard these particular stories before, they were likely highly familiar with the genre and were likely able to predict upcoming speech robustly in such continuous speech. This might have reduced the effect of noise in video communication and made listening and understanding less effortful, leading to similar levels of cortical speech tracking in both conditions. This can be further supported by the non-significant result that peak prediction accuracy in the video condition was reached with no time delay between the speech and the neural signal. As suggested in several previous studies^41,42^, it is possible that cortical speech tracking was facilitated by predictions, such that cortical activity is adapted for better efficiency based on the anticipated inputs^42^.

It is also possible that the naturalistic and social setting of our study contributed to the similar levels of cortical speech tracking in both conditions, potentially due to social affordances such as bidirectional interaction and familiar social cues. Although the task primarily required listening, the child and the experimenter were able to make eye contact and exchange information about each other’s state, at least through nonverbal cues. Since both conditions involved a live storyteller interacting with the child, these social elements may have helped mitigate the effects of the noise present in the video condition. The social and bidirectional interaction and engagement with the storyteller may have enhanced the children’s ability to track speech in both conditions, leading to successful listening and understanding with less attention and effort in the video condition. The relatively high (83%) and comparable levels of correct responses to simple comprehension questions suggest that communication was as effective in the online video condition as in the face-to-face setting. However, while highly speculative given the scarcity of available direct evidence, our findings highlight the importance of considering the social context in which communication takes place and how it may influence neural processing of speech.

The developmental trajectory of cortical speech tracking, particularly the role of audio-visual integration, remains an open question. While studies have demonstrated that infants exhibit enhanced cortical speech tracking for infant-directed speech (IDS)^22–25^, another study with 6- to 9-year-old children suggested that cortical speech tracking in noisy situations is still developing during these ages^26^. In particular, a study found that while both infants and adults showed improved cortical speech tracking when provided with audiovisual speech cues, 4-year-olds did not^25^. Although the sample in the current study is close to the age of sample in Tan et al.^25^, the current study found evidence for cortical speech tracking in a similar age-group. One possible factor contributing to the different results might be that in Tan et al., the children listened to stimuli in IDS, which might have been less interesting for 4-year-olds, thereby decreasing attention to the signal. Nevertheless, these findings highlight the need for further research into how children’s reliance on audiovisual information develops and whether attentional, linguistic, or social factors influence cortical speech tracking across different ages in different media of communication.

It is also worth noting that the prediction accuracy values reported in this study are relatively low, which may be due to several factors. First, it is difficult for children at the age range of our sample to sit relatively still for longer times, which leads to a lower signal-to-noise ratio in our EEG data. Additionally, our method of training the TRF models may have contributed to the smaller prediction accuracies reported. Specifically, we used the data from both conditions to train a single model for each participant, and tested model success separately for each condition using the same model. Although this approach may have resulted in lower prediction accuracies compared to training separate models for each condition and testing model success using trials from that condition only, we chose this more conservative approach for the sake of generalizability. Furthermore, we used data from all the channels to train the TRFs, which potentially decreases the prediction accuracies further. In future studies, contributions of individual brain regions to cortical speech tracking can be analyzed further using data with better spatial resolution.

Although this study aimed higher ecological validity, it is important to note the challenges that come with it. Using whole stories instead of short, single sentence stimuli require children to sit wearing EEG caps for a long time, which might introduce fatigue effects. Also, such stimuli might introduce other confounds, such that while some children might have found the stories particularly interesting, others might have become bored more quickly. Another source of confound are the experimenters, who, likely, introduced small variations in how they read the stories to different children. Similarly, due to our focus on the use of naturalistic settings, we used a publicly available, open-source video conferencing tool in real time. It was, therefore, not possible to ensure that quality of connection was the same all the time. Although the experimenters did not report any particular problem with the connection, it is possible that minor fluctuations in the quality might have affected children’s processing of the input.

Despite the inherent limitations, evidence from naturalistic settings, such as that provided in this study, may help to bridge the gap between highly controlled lab studies and more applied research, such as educational research, by providing a more nuanced understanding of how children interact with digital media in real-world contexts. Indeed, our task resembles children’s daily experiences with video communication such as shared book reading with a family member over platforms like Zoom or Skype or listening to a teacher in a virtual classroom. Given that cortical speech tracking has been shown to support speech processing and comprehension^43–47^, our findings can be taken to imply that the basic neural mechanisms supporting speech processing and, possibly, language learning remain robust against the less than ideal listening conditions in online video communication. However, communication (especially video communication) entails more than cortical speech tracking and efficacy of this medium in terms of language processing and learning needs broader considerations to reach more conclusive evidence. Our study, despite its narrow scope and limitations, is one of the first to investigate children’s speech processing during their interactions with others via an online video platform from a neuroscientific perspective and finds no evidence for a difference in cortical speech tracking in online versus f2f interactions. This is promising for the increasingly digital world that children are growing up in.

## Methods

### Participants

A total of 50 children participated in our study ($\hbox {Median}_{age}$ = 66.5, $\hbox {Min}_{age}$ = 60, $\hbox {Max}_{age}$ = 71 (in months); female = 20). 21 children were excluded from analysis due to fussiness (*n* = 4), not wearing EEG cap (*n* = 6), technical problems (*n* = 5), incomplete data (*n* = 4), and experimenter error (*n* = 2). This resulted in 29 children ($\hbox {Median}_{age}$ = 67, $\hbox {Min}_{age}$ = 60, $\hbox {Max}_{age}$ = 71 (in months); female = 11) whose EEG data were included in the final analyses. Based on parental reports when children were added to the database, verified prior to participation in the study, all participants were typically developing and had normal hearing and normal or corrected-to-normal sight. Children were recruited through the database of the institute through phone calls to parents who previously stated their interest in taking part in language and developmental psychology studies. All children were native speakers of German. Before the data collection started, parents and children were informed about the procedures and the parents signed an informed consent as well. All the procedures performed throughout the study were in accordance with the Declaration of Helsinki^48^, and approved by the ethics committee of Institute of Psychology of University of Goettingen, Germany.

### Stimuli

Stimuli consisted of two child-friendly and age-appropriate stories. One story was about fairies who were trying to make a magic potion to bring spring. In the other one, a few children in a kindergarten were pretending to be in a jungle. Each story took around 10 minutes to be read. A total of six experimenters took part in the study. For each participant, two experimenters were assigned based on availability﻿—one to read the story in the f2f condition and the other in the video condition. Across participants, the experimenters alternated roles to ensure a balanced distribution of readings across conditions. All experimenters were only instructed to read the stories in a natural, child-friendly manner, without any additional guidance or constraints. None of the children had heard the stories before. To maintain the attention of the children to the stories, related cartoonish pictures were shown on a 24” touch screen in front of the children. The pictures were generated by AI based on an excerpt from the stories and modified by a student assistant to be more child friendly. The picture on the screen changed a few times in each story. Children were also asked to answer between two to three comprehension questions about each story at certain points during the stories. The questions were read by the experimenter who also read the stories to the children. Children provided their answers by tapping on one of the four pictures on the touch screen. These were very simple questions about the events in the story and served the purpose of keeping the children engaged with the task. Pictures and questions appeared at the same points across all participants. Data from 500 ms before and 500 ms after the picture changes and the presentation and answering of questions were also excluded from the analyses.

### Procedure

Children and parents were welcomed in a separate room and informed about the study and the procedures. Children were shown the EEG cap on a teddy bear and told that they would listen to two stories. After parents signed the informed consent, the child, parent, and two experimenters went to the lab and one of the experimenters put the EEG cap and electrodes on the child’s head while the other played a board game with the child. The children were told that they would listen to some stories and they would be asked a few questions while they were listening. Once capping was completed, the child sat approximately 50 cm away from the touch screen, which was tilted at an angle of approximately 30 degrees to the back so that the child and the experimenter could see one another. In the f2f condition, one of the experimenters sat at the other end of the 60 by 60 cm table and began reading the story once the second experimenter started the experiment program and left the room. Pictures and questions were controlled by the experimenter via a keyboard. In the video condition, the experimenter in the room connected to a different experimenter on an 9.7” iPad via the video conferencing tool BigBlueButton^49^. The tablet was then placed on a holding arm which stood above the touch screen, around the middle of the table. This set-up allowed the child to easily see the experimenter on the iPad and the content of the touch screen at the same time. Following a sound check, once the child approved the sound level the experimenter started reading the story to the child. The sound levels were not controlled further. The pictures and questions were controlled by the experimenter, who was in the same room as the child, using the keyboard. After the first story ended, the child was told that he or she could take a break and the second story would start when they were ready. The experiment was terminated whenever the child or the parent requested to stop or when the experimenter felt that the child was becoming restless. Although all children heard the stories in the same fixed order (e.g., first fairies, then jungle kindergarten), the condition in which each story was presented (face-to-face or video) was randomized and counterbalanced across participants. This ensured that each story appeared equally often in both the face-to-face and video conditions.

Although we did not make any quantitative comparisons of audio-visual quality between the conditions, it is important to note that video conferencing tools such as BigBlueButton operate within technical constraints that can reduce signal fidelity. Specifically, audio was transmitted at a maximum of approximately 40 Kbps per direction^49^, which is substantially lower than the full-spectrum, high-fidelity signal available in face-to-face conversation. This level of compression can introduce subtle distortions and reduce the richness of prosodic cues. Additionally, the webcam resolution was likely limited to 1280$\times$ 720 pixels (approx. 0.6 Mbit/sec per stream)^49^ , a rate significantly lower than the 5-8 Mbit/sec typically recommended for smooth 1080p HD video streaming^50^. Moreover, fluctuations in connection quality-such as packet loss, jitter, and latency-can lead to temporary reductions in both audio and video quality, including freezing, desynchronisation, or compression artefacts. Thus, even under optimal conditions, online video in our experiment did not provide the same consistent and high-quality sensory input as face-to-face interaction.

Audio was recorded throughout the experiment using Audio-Technica 2020 microphone at 48 kHz sampling rate with 16-bit resolution. The microphone was placed between the experimenter and the participant. For the video condition, the same microphone was used instead of recording the audio from the video conferencing tool to capture the exact listening conditions of the participant.

We also recorded videos of children which were used to check if there were any problems in the session. We also sent a comprehensive questionnaire about their children’s and family members’ use of digital devices for another study, so these responses were not analysed for this study.

### EEG data

EEG was recorded using BioSemi Active Two amplifier at 2048 Hz sampling rate. Children wore caps with 32 active electrodes in 10-20 international convention layout. Our BioSemi system uses, instead of a conventional online reference channel, a Common Mode Sense (CMS) active electrode to monitor the average voltage of all electrodes on the scalp and a Driven Right Leg (DRL) passive electrode to actively counteract the common mode voltage detected by the CMS. In total 32 channels were recorded: Fp1, AF3, F7, F3, FC1, FC5, T7, C3, CP1, CP5, P7, P3, Pz, PO3, O1, Oz, O2, PO4, P4, P8, CP6, CP2, C4, T8, FC6, FC2, F4, F8, AF4, Fp2, Fz, Cz. Electrode impedances were kept below 50 k$\Omega$. Two additional electrodes were placed on the mastoids and one under left eye to monitor muscle artefacts. All recordings and stimulus presentation (pictures and questions) were controlled by the same Python script and the synchronisation between EEG data and audio recordings was established via triggers.

#### Pre-processing

EEG data were pre-processed using the EEGLAB toolbox (version 2023.0)^51^ on MATLAB R2018a^52^. Following the recent discussion in the literature questioning the effectiveness of several steps of EEG pre-processing^53^ and the robustness of TRF analysis to the noise^38^, we kept pre-processing to a minimum. However, we reran the analyses with different pre-processing pathways to ensure that the results are not an artefact of the pre-processing strategy. For this, we used The Harward Automated Processing Pipeline in Low Density Electroencephalography (HAPPILEE)^54^ script. These additional analyses produced results with similar patterns and are reported in supplementary material B.

First, the raw data outputted by BioSemi (.bdf files) were loaded into MATLAB environment using the mastoids as reference electrodes. Two mastoids and one channel for the eye-movements were then removed from the data set, resulting in data from 32 channels. As we did not have hypotheses about specific frequency bands, the data were high pass filtered at 0.1 Hz and low-pass filtered with 40 Hz cut-offs using Butterworth 12th order filters, following the similar applications in the literature^40,55^. The filtering was carried out in both directions to prevent phase shifts. The EEG data was then downsampled to 128 Hz to make subsequent processing more efficient.

The audio recordings were also imported to the MATLAB environment, resampled to the same sampling rate as the EEG data, i.e., 128 Hz. The speech envelope is extracted using mTRF toolbox (version 2.2)^40^, by logarithmically scaling the root mean square intensity. In the next step, EEG and speech envelope were synchronised. Both time series data were synchronised using the trigger that signaled the start of audio recording in EEG data. Data recorded while the picture on the screen was changing and the questions were presented was excluded from further analysis. This process split the EEG and speech envelope data into 14 chunks with unequal length. These chunks were not concatenated to avoid introducing further artefacts. Instead, for each participant, the minimum chunk duration was determined and each chunk was divided further into smaller chunks, each with the length of the smallest full chunk. Although this caused a further 20% data loss, it allowed each participant to have a number of equal-length “trials”, which could then be used to train and test the TRF models, and prevented artefacts related to concatenation of time series. At the end, there were on average 14.8 trials in f2f ($\hbox {sd}_{trial\_no\_f2f}$ = 2.06) and 16.1 trials in video condition ($\hbox {sd}_{trial\_no\_video}$ = 2.57) with an average trial duration of 28.5 seconds ($\hbox {sd}_{trial\_dur}$ = 2.69) for each participant.

### Data analysis

Cortical speech tracking was operationalized by modelling a response function that maps a linear relationship from the stimulus speech envelope to the neural response using mTRF Toolbox^40^. We fitted a forward model, which ascertains the optimal weights to predict the neural signal from the stimulus features, i.e., the speech envelope in this case. The weights were determined through a lagged ridge regression, which takes the possible delay between the stimulus onset and emergence of processing markers in the neural signal. These weights are biologically meaningful and protect the direction of causality, i.e., significant non-zero weights capture the relationship between the stimulus and the neural response^56,57^. After TRF weights were obtained, they were used to generate neural signals based on a segment of speech envelope, which was not used in training the model to obtain the weights. The correlation between the predicted neural signal and the actual neural signal is interpreted as prediction accuracy and used as a measure of cortical speech tracking; higher prediction accuracies are interpreted as better cortical speech tracking.

In the literature, testing the model, i.e., predicting the neural signal from the stimuli that were not used in model training, is achieved either at the subject level or at the group level. At the subject level testing, the model is trained with $\textrm{n}-1$ trials from each subject and then tested using the stimulus and the response of nth trial of the same participant^58,59^. In the group level evaluation, average model weights are obtained for n-1 participants and then the model is tested using the data from $\hbox {n}^{th}$ participant (e.g., Jessen et al.,^58^; Tan et al.,^25^). As in our study each subject provided slightly different durations of data and each heard the stories with some variation (e.g., different experimenters reading the stories), we used a subject-dependent approach.

We started by identifying the optimal regularization parameter using Tikhonov regularization, which penalizes large coefficients to prevent overfitting the model to the noise and increase the generalizability of model fitting to new data^40^. Thus, we ran a leave-one-out cross validation to find the optimal regularization value ($\lambda$) for each participant. Optimal $\lambda$ was ascertained from a set of values ranging from $10^{-11}$ to $10^{11}$ with an increase at the order of $10^{2}$ at each step. To determine the optimal $\lambda$ on a larger data set, this step was conducted before the data were split into trials.

After determining optimal regularization parameter, one TRF model was trained on all trials for each participant in a leave-one-out manner. Based on the previous literature^38,58^ and our pre-registration, a time delay between 0 and 600 ms was determined and entered as one of the model parameters. The model training was iterated over the trials and in each iteration one trial was omitted from the training data set. For each iteration, the resulting model was then used to predict a neural signal from the speech envelope of the omitted trial. Model success was evaluated by applying Pearson’s correlation between the actual neural signal and the predicted signal and correlation coefficients for trials from different conditions were stored separately. The resulting Pearson’s correlation coefficients were used as our measure of “prediction accuracy” in subsequent analyses.

Previous studies typically examine topographical maps of TRF weights and prediction accuracies to ascertain each recording channel’s individual contribution and choose channels for further analyses based on how much they contribute to prediction accuracy. However, given that we did not include specific hypotheses about differing contributions of different scalp regions to the observed effects and the low spatial density of our recording (32 channels), we included the data from all the available channels in our final analyses. We also averaged the TRF weights and prediction accuracies across the entire scalp. However, topographical maps are included in supplementary material C.

We conducted a permutation analysis on the available data to test whether cortical speech tracking in both conditions is significantly better compared to a random distribution (﻿1). We ran 1000 permutations where, in each iteration, the neural signal of each trial for each participant was randomly paired with the speech envelope of another trial from the same participant. One iteration was run without shuffling the data to represent the actual prediction accuracies in the random distribution as well. Then, the model training and model evaluation was conducted in the same way as described above. This produced 1000 correlation coefficients for each trial of each participant. We then averaged these coefficients over trials and participants to obtain in total 1000 correlation coefficients which show the cortical tracking of a random speech envelope. We compared our actual correlation coefficients with the results of the permutation to determine significance using the equation (1).

1 $$\begin{aligned} \begin{aligned} p = \frac{\sum I(R_i \ge A)}{N} \end{aligned} \end{aligned}$$

where:

- $I(R_i \ge A)$ ) is an indicator function that equals to 1 if the prediction accuracy ($R_i$) is greater than or equal to the actual prediction accuracy (*A*), and 0 otherwise.
- *N* is the total number of prediction accuracies obtained from permutations

A generalized linear mixed effects model with beta distribution (Eq. 2) was used to test whether speech prediction was more successful in the video condition relative to the f2f condition (H.2.1.) and whether there were temporal differences in terms of when the maximum prediction accuracy emerges between the two conditions (H.2.2.). The response was the correlation coefficients between predicted and actual neural signal. Condition (factor with two levels: f2f vs. video), time delay (numeric, ranging between 0 and 600), and time delay squared were entered as predictors. Two-way interactions between condition and time delay and condition and time delay squared were included as well. Time delay squared was added to the model to examine a possible non-linear relationship between prediction accuracy and time delay. That is, it is possible that prediction accuracy increases with time delay up to a certain point (e.g. 300 ms. delay) and decreases after the peak. We also included random intercepts of participant ID and random slopes of the main effects and two-way interactions between condition, time delay, and time delay squared within participant ID. Correlations between random intercept and slopes were initially included, but whenever the models were not successfully fitted, we compared the log likelihood of each model including the correlations and not including the correlations and dropped the correlations if the difference was small. Furthermore, we dropped correlation parameters when they were close to $-1$ or 1 and their exclusion led to an only minor decrease in model fit. The final model was formulated using glmmTMB function of glmmTMB package^60^ in R, version 4.2.2^61^ (R Core Team, 2019).

2 $$\begin{aligned} \begin{aligned} {pred\_accuracy} \sim {condition} \times ({time} + {time\_squared}) + (1 + {condition} \times ({time} + {time\_squared}) \, || \, {sub\_id}) \end{aligned} \end{aligned}$$

Our data analysis diverged from what we had planned in our pre-registration in two ways. First, although we had initially planned to use backward models, we chose forward models instead, as they offer more interpretable results—﻿TRF weights in forward models reflect how the brain responds to the speech signal, while weights in backward models are not directly interpretable^40^. Second, we dropped a few steps of pre-processing in the current analysis. However, the pre-registered analyses conducted on the same data are presented in supplementary material D, and they suggest similar results.

## Data Availability

The datasets generated and analysed during the current study are not publicly available due to inherent identifiability in EEG and speech data but can be made available to be processed on the servers of University of Göttingen upon request. Requests for access to data can be sent to Fatih Sivridag (fatih.sivridag@uni-goettingen.de).
